# Supplementary material for: Tree growth is better explained by absorptive fine root traits than by transport fine root traits
Source: Commun Biol. 2025 Feb 26;8:313. doi: 10.1038/s42003-025-07756-y (PMC11865288; doi:10.1038/s42003-025-07756-y)
Supplement: Supplementary file 1 — Supplementary Information [file 42003_2025_7756_MOESM1_ESM.pdf]

Supplementary Information for:

Tree growth is better explained by absorptive fine root traits than by transport fine root traits

Anvar Sanaei[1*], Fons van der Plas[1,2], Hongmei Chen[1,3], Sophie Davids[1], Susanne Eckhardt[1], Justus Hennecke[1,4], Anja Kahl[1], Yasmin Möller[1], Ronny Richter[1,4], Jana Schütze[1], Christian Wirth[1,4,5], Alexandra Weigelt[1,4]

[1] Institute of Biology, Leipzig University, 04103 Leipzig, Germany

[2] Plant Ecology and Nature Conservation Group, Wageningen University, P.O. Box 47, Wageningen, The Netherlands

[3] Lancaster Environment Centre, Lancaster University, Lancaster LA1 4YQ, UK

[4] German Centre for Integrative Biodiversity Research (iDiv) Halle-Jena-Leipzig, 04103 Leipzig, Germany

[5] Max-Planck-Institute for Biogeochemistry, 07745 Jena, Germany

Table S1. Results of the principal component analysis (PCA) based on the correlation matrix of 25 broadleaved tree species for fine roots (absorptive and transport roots) and leaf traits as shown in Figure 3. SRL is specific root length; RTD is root tissue density; RD is average root diameter; C:S is cortex to stele ratio; MCR is mycorrhizal colonization rate; LDMC is leaf dry matter content; LMA is leaf mass per area; and LT is leaf toughness. Displayed data are the variance explained by each principal component (PC) and the loading scores of the root and leaf traits on the first four PC axes. Following Figure 3e, the loading scores of the first and second PC axes for transport root and leaf traits are inverted.

Absorptive root traits					*Transport root traits*				
**Fig. 3a**	**PC1**	**PC2**	**PC3**	**PC4**	**Fig. 3b**	**PC1**	**PC2**	**PC3**	**PC4**
Variance	0.531	0.254	0.156	0.047	Variance	0.348	0.317	0.193	0.082
SRL	-0.46	0.55	0.03	0.37		-0.69	-0.12	-0.02	-0.10
RTD	-0.23	-0.80	0.21	0.10		0.44	-0.30	-0.66	0.33
RD	0.57	0.12	-0.24	-0.45		0.56	0.16	0.56	-0.19
MCR	0.55	-0.10	-0.19	0.81		0.06	0.62	-0.50	-0.60
C:S	0.33	0.18	0.93	0.01		-0.11	0.70	0.03	0.70

Leaf traits			
**Fig. 3c**	**PC1**	**PC2**	**PC3**
Variance	0.572	0.313	0.115
LDMC	-0.69	0.06	0.72
LMA	-0.62	0.46	-0.64
LT	-0.37	-0.89	-0.27

Absorptive root and leaf traits					*Transport root and leaf traits*				
**Fig. 3d**	**PC1**	**PC2**	**PC3**	**PC4**	**Fig. 3e**	**PC1**	**PC2**	**PC3**	**PC4**
Variance	0.410	0.209	0.122	0.104	Variance	0.299	0.216	0.175	0.123
SRL	-0.28	0.64	0.01	0.04		0.19	0.65	0.05	-0.05
RTD	-0.30	-0.45	0.18	-0.01		-0.37	-0.21	-0.47	-0.35
RD	0.48	-0.19	0.14	-0.13		-0.09	-0.59	0.12	0.48
MCR	0.45	-0.29	-0.10	-0.23		0.41	-0.32	-0.17	-0.51
C:S	0.32	-0.01	0.37	0.77		0.36	-0.23	0.41	-0.47
LDMC	-0.42	-0.31	-0.21	-0.13		-0.54	0.12	0.17	-0.34
LMA	-0.29	-0.35	-0.39	0.53		-0.47	-0.03	0.14	-0.22
LT	-0.19	-0.3	-0.78	-0.18		-0.12	-0.06	0.72	-0.04

Table S2. Results of the principal component analysis (PCA) based on the correlation matrix of 25 broadleaved tree species for fine roots (absorptive and transport roots) and leaf traits as shown in Figure S2. SRL is specific root length; RTD is root tissue density; RD is average root diameter; C:S is cortex to stele ratio; MCR is mycorrhizal colonization rate; LDMC is leaf dry matter content; LMA is leaf mass per area; and LT is leaf toughness. Displayed data are the variance explained by each principal component (PC) and the loading scores of the root and leaf traits on the first four PC axes. Absorptive traits are ended with an **A** letter, while transport roots are ended with a **T** letter.

Absorptive and transport root traits				
**Fig. S2a**	**PC1**	**PC2**	**PC3**	**PC4**
Variance	0.40	0.253	0.122	0.086
SRL_T	-0.20	-0.44	-0.25	-0.18
SRL_A	-0.39	-0.35	0.03	0.05
RTD_T	-0.02	0.47	-0.46	-0.14
RTD_A	-0.14	0.48	-0.09	-0.49
RD_T	0.15	0.36	0.61	0.14
RD_A	0.45	-0.08	0.08	0.32
MCR_T	0.45	-0.08	-0.32	0.01
MCR_A	0.44	-0.03	-0.38	0.08
C:S_T	0.30	-0.23	0.22	-0.40
C:S_A	0.26	-0.18	0.23	-0.65
Root (absorptive and transport roots) and leaf traits				
**Fig. S2b**	**PC1**	**PC2**	**PC3**	**PC4**
Variance	0.342	0.219	0.116	0.088
SRL_T	-0.11	-0.45	-0.11	-0.25
SRL_A	-0.29	-0.44	-0.01	0.05
RTD_T	-0.10	0.41	-0.44	-0.12
RTD_A	-0.21	0.39	0.01	-0.09
RD_T	0.09	0.35	0.33	0.54
RD_A	0.43	0.06	0.01	0.06
MCR_T	0.43	0.06	-0.18	-0.27
MCR_A	0.41	0.09	-0.24	-0.27
C:S_T	0.29	-0.07	0.40	-0.36
C:S_A	0.27	-0.09	0.23	0.01
LDMC	-0.31	0.24	0.01	-0.44
LMA	-0.21	0.26	0.13	-0.21
LT	-0.10	0.08	0.60	-0.33

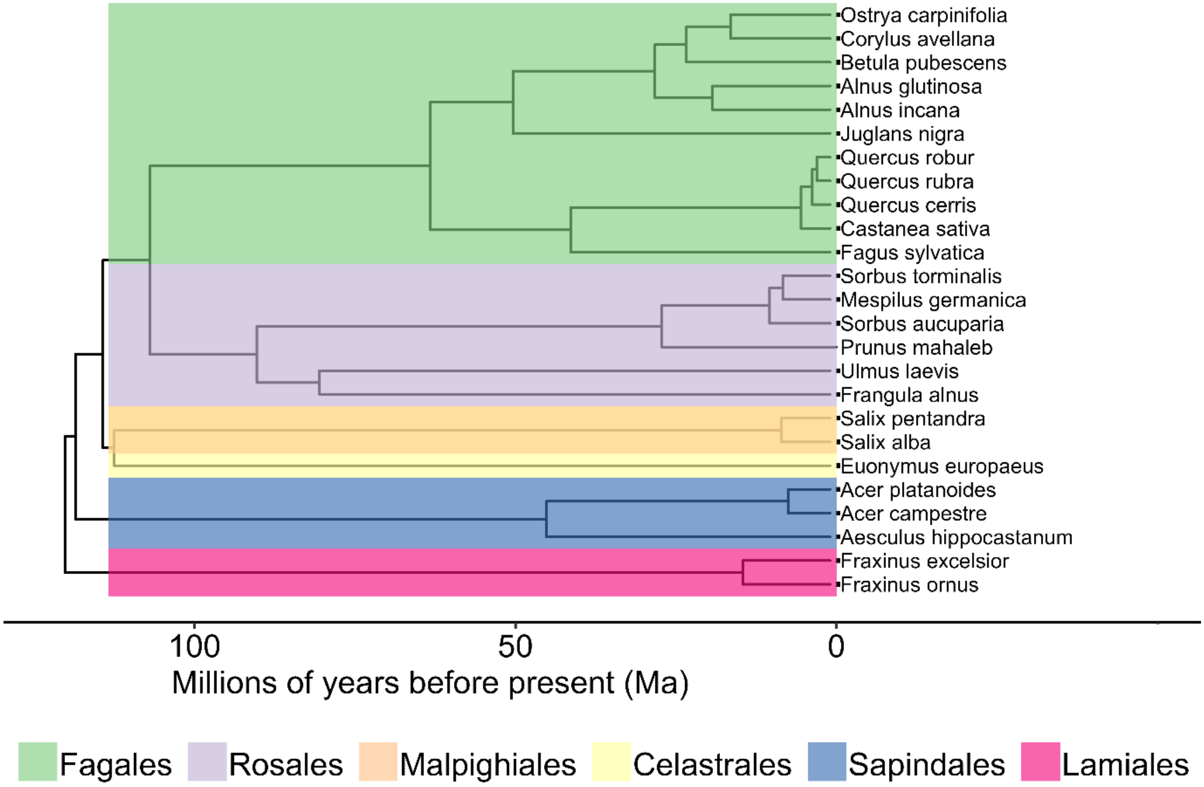

Figure S1. Phylogenetic tree of the 25 broadleaved tree species included in this study. Different orders are shown with different colors.

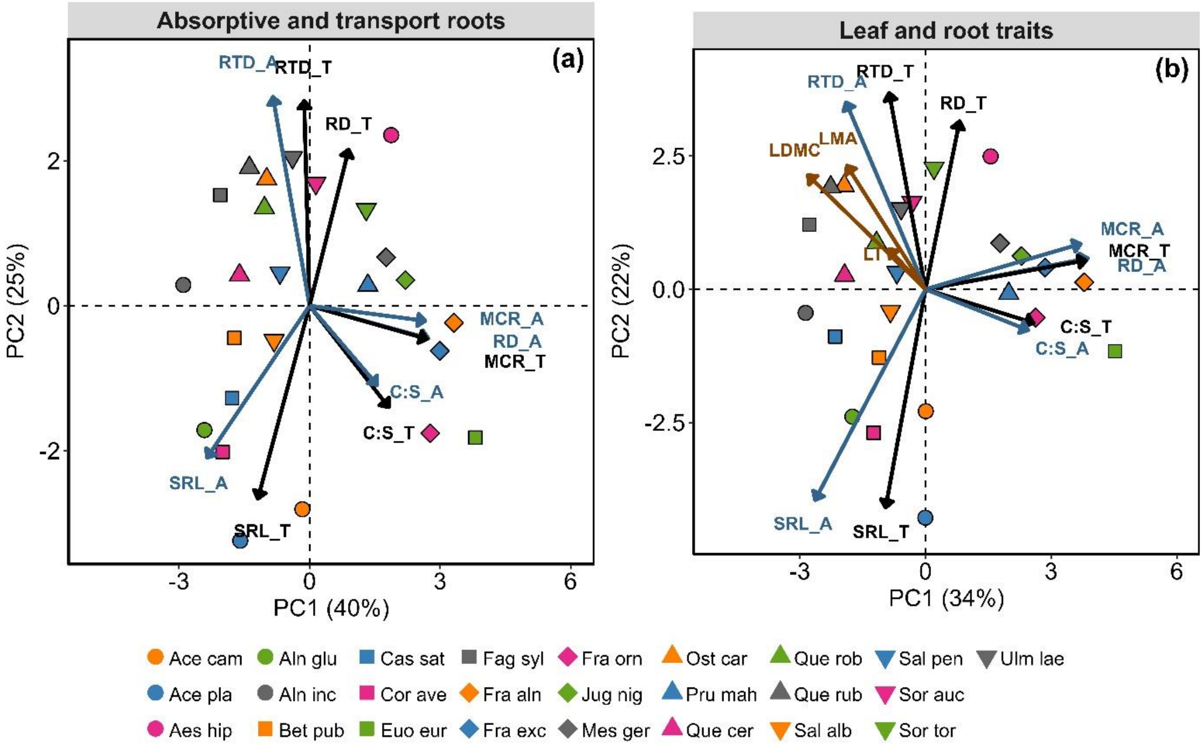

Figure S2. Principal component analyses (PCA) of species-levels of (a) absorptive and transport root traits, and (b) the whole set of root (absorptive and transport roots) and leaf traits. Abbreviations for traits are as follows: RD, root diameter; RTD, root tissue density; SRL, specific root length; C:S, cortex to stele ratio; MCR, mycorrhizal colonization rate; LMA, leaf mass per area; LT, leaf toughness; LDMC, leaf dry matter content. Absorptive traits (ending with an A letter) are shown with black arrows, while transport root traits (ending with a T letter) are shown with light blue arrows and leaf traits are shown with brown arrows. Different point colors and shapes represent different tree species. See Table 1 and Figure S1 for tree species name abbreviations.

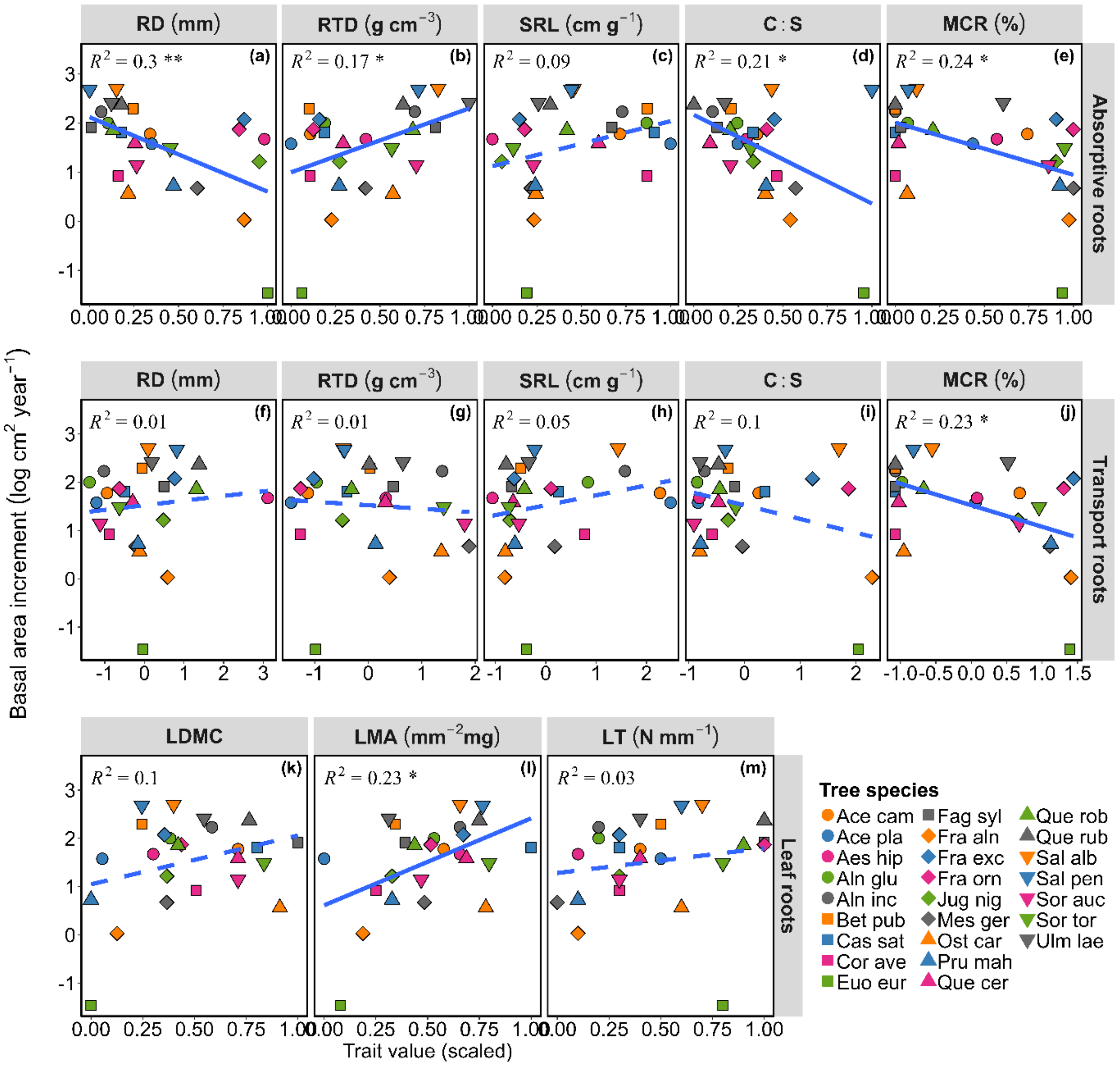

Figure S3. The relationships between a:e) absorptive root traits, and f:j) transport root traits, and k:m) leaf traits with average basal area increment. Average basal area increment data were log-transformed in regression, while root and leaf trait data were scaled. Significant correlations are indicated as solid lines and non-significant relationships as dashed lines, and the shaded areas denote the 95% confidence interval. Shown are the R^2 of the regressions, which refers to the proportion of variance in basal area increment explained by root and leaf traits. Significant relationships between basal area increment and root or leaf traits are denoted by * (*P < 0.05*), ** (*P < 0.01*) and *** (*P < 0.001*). Trait abbreviations follow the caption in Figure S2. Different point colors and shapes represent different tree species. See Table 1 and Figure S1 for tree species name abbreviations.

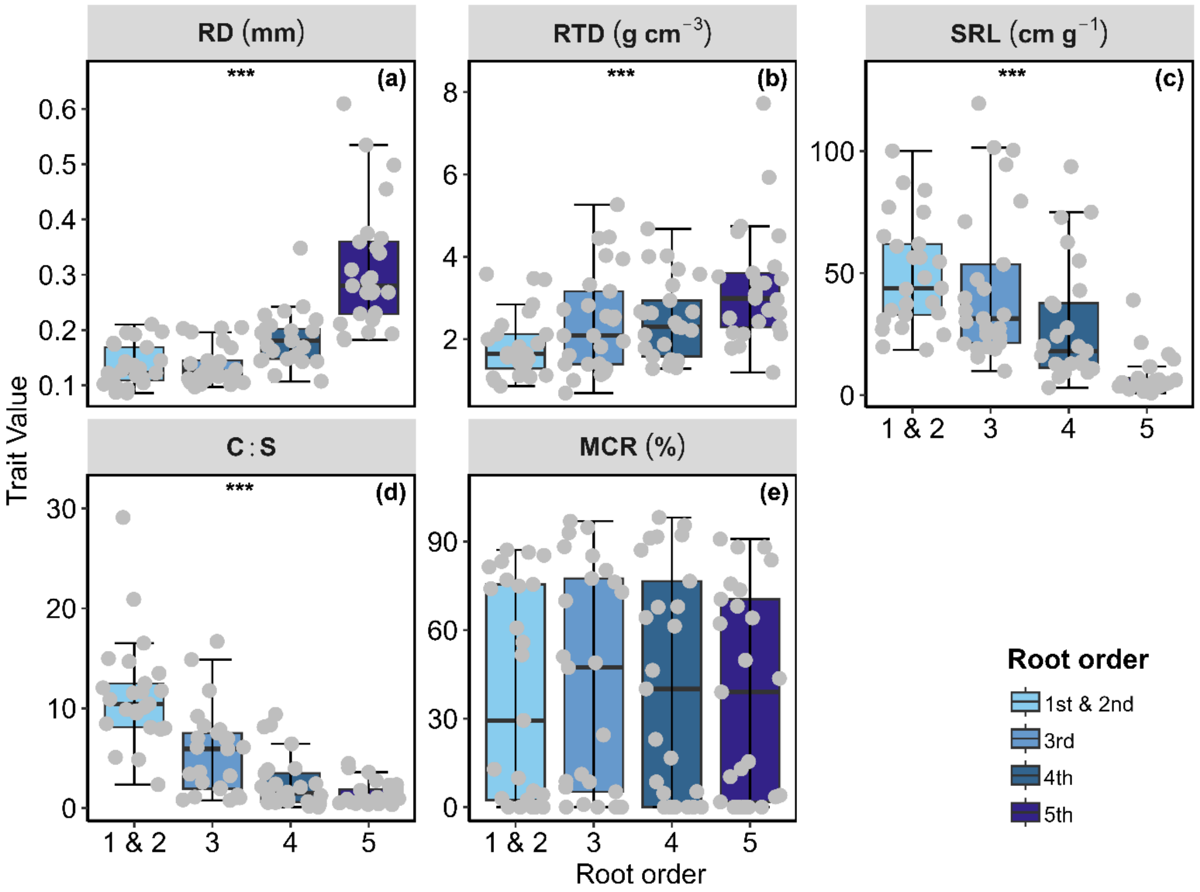

Figure S4. Changes in root traits across root orders. Significant differences across root orders are denoted by * (*P < 0.05*), ** (*P < 0.01*) and *** (*P < 0.001*). Data represented are the median (line in the middle) ± IQR. Trait abbreviations follow the caption in Figure S2. Samples are colored according to root order.

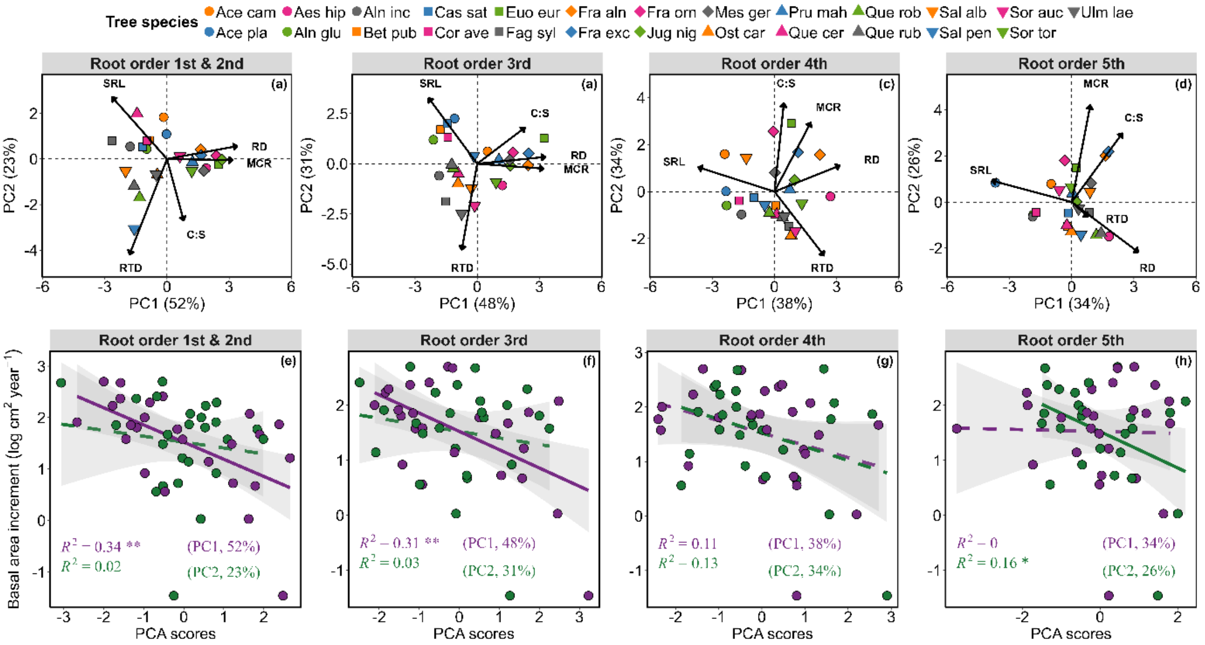

Figure S5. Principal component analyses (PCAs) of root traits across root orders (a-d), and the relationships between the principal component (PC) axes of root traits and average basal area increment (e-h). Shown are the R^2 values of the linear regressions. Significant relationships between basal area increment and PC axes are denoted by * ($P < 0.05$), ** ($P < 0.01$) and *** ($P < 0.001$). Trait abbreviations follow the caption in Figure S2. Different point colors and shapes (a-d) represent different tree species, while points (e-h) are colored to distinguish PC1 (purple) from PC2 (green). See Table 1 and Figure S1 for tree species name abbreviations. Note that the second PC axis of the root order 3rd (b,f) and the first and second PC axes of the root order 5th (d,h) are inverted.

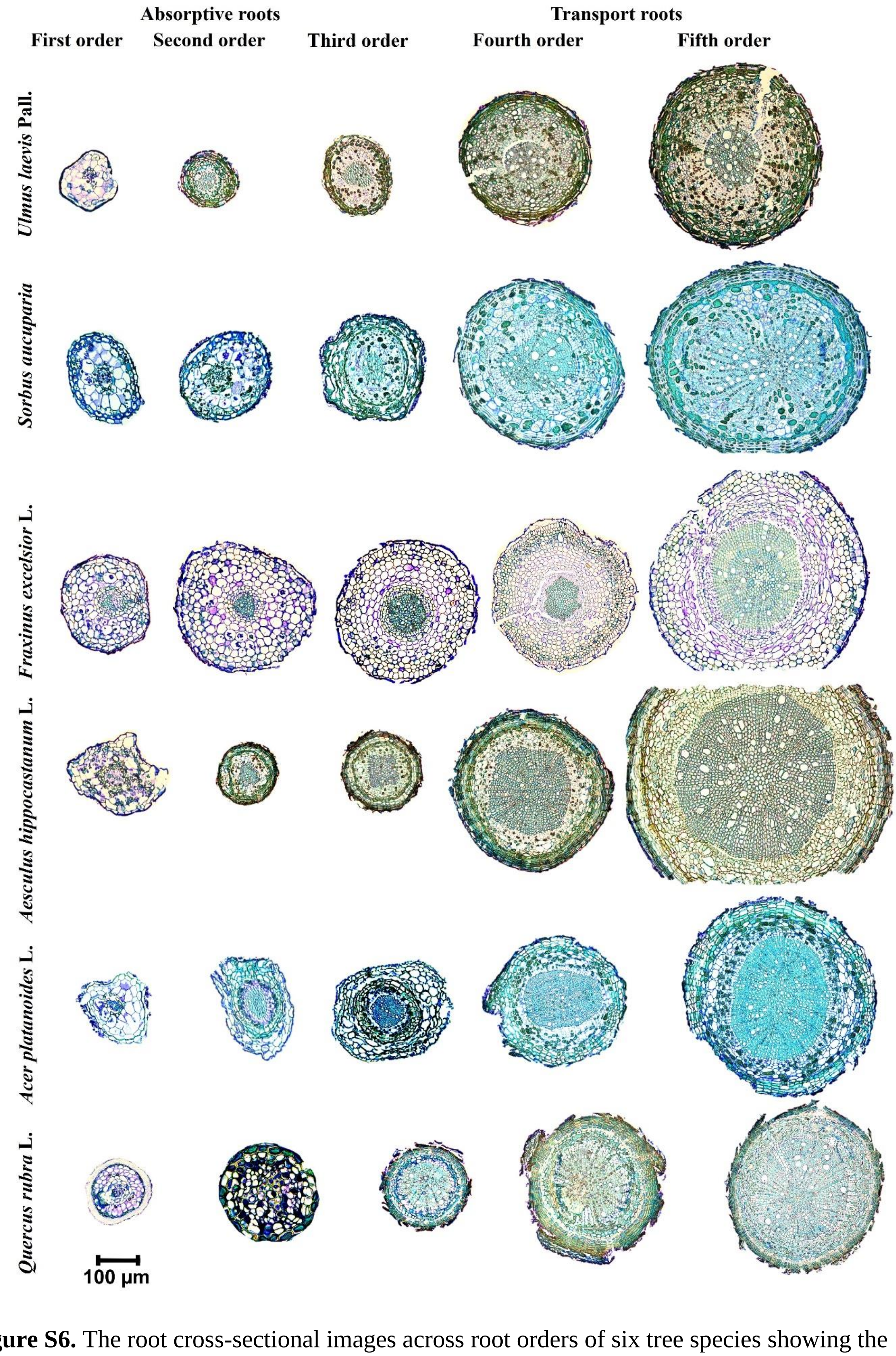

Figure S6. The root cross-sectional images across root orders of six tree species showing the distinct variations of anatomical structures across root orders at magnification of 10x (100 µm).
